# Alternative Clinical Careers for Physicians: A Mentor-Focused Study of Medical Science Liaison Career Awareness During a Wilderness Medicine Program in Chamonix-Mont-Blanc, France

**DOI:** 10.7759/cureus.109427

**Published:** 2026-05-22

**Authors:** Samuel J Dyer, Richard Ingebretsen, Jeff Kraemer

**Affiliations:** 1 Medicine, Medical Science Liaison Society, Miami, USA; 2 Medicine, University of Utah, Salt Lake City, USA; 3 Medical Affairs, Medical Science Liaison Society, Miami, USA

**Keywords:** alternative clinical careers, career awareness, emergency medicine and trauma, medical affairs, medical education, medical science liaison, mentorship, pharmaceutical industry, wilderness medicine

## Abstract

Introduction

Medical education traditionally emphasizes direct patient care within established clinical specialties, with limited structured exposure to alternative clinical careers. Increasing numbers of medical students and residents are exploring non-traditional physician pathways; however, formal curricular exposure to roles in the pharmaceutical industry is limited. This descriptive study evaluated whether structured mentor-focused sessions within the University of Utah School of Medicine Wilderness Medicine Program influenced medical students' and residents' awareness of and interest in careers within the pharmaceutical industry, particularly the Medical Science Liaison (MSL) profession.

Methods

This descriptive pre- and post-session survey study was conducted during the 2025 University of Utah School of Medicine Wilderness Medicine Program in Chamonix-Mont-Blanc, France. The program included 65 undergraduate participants and 40 medical students and residents from diverse institutions and six countries. Medical students and residents who voluntarily attended structured mentor-focused sessions on alternative clinical careers within Medical Affairs, particularly the MSL profession, were eligible to participate. The educational intervention consisted of two interactive mentor-led sessions that included didactic presentations and moderated discussions on physician roles within the pharmaceutical industry. Anonymous electronic surveys consisting of single-response multiple-choice and Likert-type questions were administered immediately before and after the sessions using quick response (QR) code access.

Results

Nineteen participants completed the pre-session survey, and 11 completed the post-session survey, with a smaller post-session sample size and potential for response bias. Prior to the sessions, 12 (63.2%) had not previously considered a career in the pharmaceutical industry, and 11 (57.9%) indicated that medical school had not prepared them to explore such career options. Following the sessions, seven (63.6%) reported being "much more" informed about physicians' roles in the pharmaceutical industry, eight (72.7%) reported being "somewhat" or "very interested" in learning more, and six (54.5%) selected the MSL role as the most interesting career option presented.

Conclusion

Prior to the mentor sessions, most participants reported limited familiarity with physician roles within the pharmaceutical industry and limited exposure to alternative clinical career pathways during medical training. Following participation in the sessions, respondents reported increased understanding of pharmaceutical physician roles and greater interest in learning more about these careers, with the MSL role emerging as the most frequently selected role of interest. These findings suggest that even brief, structured mentor-focused educational interventions may increase the awareness of alternative physician career pathways and highlight the potential value of incorporating career exploration opportunities into medical education curricula.

## Introduction

Medical education has historically centered on preparing trainees for direct patient care within traditional clinical specialties. While this focus remains essential, the professional landscape for physicians has expanded significantly over the past several decades. Increasing numbers of medical students and residents are exploring alternative clinical career pathways that allow them to apply their medical training to non-traditional roles, including public health, health policy, consulting, and research administration, and others [1-3]. Despite this trend, structured exposure to non-traditional physician career pathways remains limited within most medical school curricula [1-5].

Among alternative clinical pathways, careers within the pharmaceutical industry have become increasingly visible. Within these industries, Medical Affairs departments serve as the scientific interface between pharmaceutical organizations and the healthcare community. Physician roles within Medical Affairs include Medical Director, Clinical Development, Drug Safety/Pharmacovigilance Physician, Chief Medical Officer, Vice President of Medical Affairs, Regulatory Affairs Lead, and Medical Science Liaison (MSL) [6-11].

The responsibilities and activities of the MSL profession have expanded significantly over the past two decades in response to increasing therapeutic complexity, regulatory requirements, and demand for high-level scientific exchange [10-13]. MSLs are scientifically trained professionals, typically holding advanced degrees such as MD, PharmD, or PhD, who engage in scientific dialogue with healthcare professionals (HCPs) [10-12]. The primary purpose of the MSL is to support key opinion leaders (KOLs) and other HCPs in making more informed clinical decisions through balanced, evidence-based discussion of scientific data [11]. MSLs operate within Medical Affairs departments at pharmaceutical, biotechnology, medical device, and diagnostic companies [11,12]. Their responsibilities include scientific communication, medical education, clinical trial support, and the gathering of KOL insights that inform medical and strategic decision-making [12,13]. Unlike commercial roles such as pharmaceutical sales representatives, MSLs function within a non-promotional framework centered on scientific integrity and patient outcomes [11-14].

Despite the increasing prominence of the MSL profession globally, awareness among medical trainees remains variable [1,3,5]. Surveys conducted in Europe and North America demonstrate heterogeneity in familiarity with MSL responsibilities, training structures, and performance expectations [6,7]. Additionally, literature examining key performance indicators, tracking systems, and role evolution highlights the increasing formalization and strategic importance of Medical Affairs functions [8-10]. However, structured exposure to these roles during medical education remains inconsistent [1-5].

The University of Utah School of Medicine Wilderness Medicine Program offers a unique educational environment in which broader professional identity formation may occur [15-21]. Conducted annually in Chamonix-Mont-Blanc, France, in the French Alps, the two-week immersive program integrates didactic instruction, alpine rescue training, remote trauma simulations, glacier travel, expedition medicine principles, and leadership development in austere environments.

In 2025, the University of Utah School of Medicine Wilderness Medicine Program introduced structured mentor-focused sessions for the first time, specifically designed for medical students and residents interested in exploring alternative clinical careers within Medical Affairs in the pharmaceutical industry, including the MSL profession. These sessions were optional and conducted independently of the core wilderness medicine curriculum. The purpose of this study was to descriptively evaluate whether structured mentor sessions conducted during an immersive Wilderness Medicine program influenced medical student and resident awareness, familiarity, and interest in alternative pharmaceutical industry careers, with particular emphasis on the MSL role.

## Materials and methods

Study design

This study utilized a descriptive cross-sectional pre- and post-session survey design to evaluate changes in awareness, familiarity, and interest in pharmaceutical industry physician careers among medical students and residents participating in structured mentor sessions. Anonymous surveys were administered immediately before and after the educational sessions.

Setting

The study was conducted during the University of Utah School of Medicine Wilderness Medicine Program held in Chamonix-Mont-Blanc, France, from June 30 through July 12, 2025. The program included undergraduate participants, medical students, and residents participating in a wilderness medicine educational curriculum that combined didactic instruction with field-based experiential learning in alpine environments. The 2025 cohort included 65 undergraduate participants and 40 medical students and residents from across the United States and six countries.

Participants

Eligible participants included medical students and residents enrolled in the 2025 Wilderness Medicine program who elected to attend the optional mentor-focused sessions on alternative clinical careers within Medical Affairs in the pharmaceutical industry. Participation in the mentor sessions was voluntary. Participation in the surveys was also voluntary and anonymous. Given the optional nature of the mentor sessions and the limited number of eligible participants within the program cohort, the study was intended as an exploratory educational evaluation. A total of 19 participants completed the pre-session survey, and 11 participants completed the post-session survey. Surveys were not linked at the individual level to preserve anonymity.

Mentor session structure and content

Two structured mentor sessions were conducted during the program. The sessions were designed to provide foundational exposure to pharmaceutical industry physician roles, with emphasis on Medical Affairs and the MSL profession.

The mentor sessions included an overview of the pharmaceutical, biotechnology, medical device, and diagnostic industries, as well as the role of Medical Affairs departments within pharmaceutical organizations. Participants were introduced to various physician career pathways, including Chief Medical Officer, Medical Director, Clinical Development, Drug Safety/Pharmacovigilance, Regulatory Affairs, and MSL roles. The sessions also included a detailed discussion of the MSL profession, including scientific exchange principles, non-promotional compliance frameworks, evidence-based communication, and global field engagement responsibilities. Additional topics included performance metrics, benchmarking systems, evolving expectations within Medical Affairs, and broader global trends influencing the field, such as digital transformation and data integration. The sessions were designed to provide balanced, informational, and non-promotional educational content regarding physician career pathways within the pharmaceutical industry. The sessions concluded with an open question-and-answer discussion focused on career pathways, qualifications, misconceptions, and professional development opportunities. Additional details regarding the educational intervention and training program are provided in the Appendices.

Survey instrument

A total of 10 survey questions, consisting of five pre-session and five post-session items, were administered. All questions were single-response, including categorical multiple-choice and Likert-type scales. No open-ended or free-text responses were collected.

Pre-session Questions Assessed

The pre-session survey assessed participants' prior consideration of pharmaceutical industry careers, familiarity with physician roles within the pharmaceutical industry, awareness of specific physician roles, likelihood of considering a pharmaceutical industry career, and perceived adequacy of medical school preparation for exploring industry-related career pathways.

Post-session Questions Assessed

The post-session survey assessed changes in participants' understanding of pharmaceutical physician roles, level of interest in learning more about pharmaceutical industry careers, perceived influence of the sessions on interest in alternative clinical careers, likelihood of seeking mentorship from professionals working within the pharmaceutical industry, and identification of the pharmaceutical physician role found to be most interesting following the sessions.

The survey instrument was developed specifically for this educational evaluation and was not previously validated. Survey questions were developed by the study authors based on the educational objectives of the mentor sessions, prior Medical Affairs and MSL educational content, and previously published literature related to physician career exploration and Medical Affairs roles [1-10]. The instrument was designed to assess perceived awareness, familiarity, and interest in pharmaceutical industry careers rather than to measure factual knowledge. Prior to administration, the survey questions were reviewed by the study authors and additional Medical Affairs subject matter experts to assess content clarity, relevance, and alignment with the educational objectives and content delivered during the sessions. The survey instrument was not pilot tested, and no formal assessment of survey reliability was performed prior to administration. The survey instrument was intended for exploratory educational evaluation and did not undergo formal psychometric validation. All survey questions and response categories are presented exactly as written in the original pre- and post-session instruments administered during the mentor sessions. No modifications to wording or response options were made between survey administration and data analysis. The tables presented in the Results section reproduce the exact question wording and response categories from the original survey instruments to ensure methodological transparency.

Data collection

Both surveys were administered electronically during the mentor sessions. A quick response (QR) code was displayed on a slide, allowing participants to access the survey on their personal devices. The pre-session survey was completed immediately prior to the first mentor session. The post-session survey was completed immediately following the second mentor session. Data were collected and analyzed using the Alchemer survey software (Version 6.9.1; Alchemer, Louisville, Colorado, United States). Responses were exported for descriptive analysis.

Data analysis

Data analysis was descriptive. Frequencies and percentages were calculated for each response category. Results are presented in tabular form for each question, grouped as Pre-Session and Post-Session. No inferential statistical testing was conducted due to the exploratory design and limited sample size.

Ethical considerations

The survey was anonymous and voluntary. No personally identifiable information was collected. Participation had no impact on academic evaluation or program standing. The study constituted an internal educational program evaluation with minimal risk and did not require institutional review board review per institutional guidance.

## Results

A total of 19 participants completed the pre-session survey, representing 47.5% of the 40 eligible medical trainees. Eleven trainees completed the post-session survey, representing 27.5% of the eligible cohort. Findings are presented descriptively to summarize baseline awareness and observed changes in understanding and interest.

Pre-session findings

The pre-session survey assessed baseline awareness, familiarity, and perceptions of physician roles in the pharmaceutical industry prior to exposure to the mentor sessions.

Before participating in the mentor sessions, consideration of a career in the pharmaceutical industry was limited. A majority of respondents indicated that they had not previously considered this as a career, while slightly more than one-third reported prior consideration (Table 1).

**Table 1:** Have you ever considered a career in the pharmaceutical industry as a physician?

Response	n	%
Yes	7	36.8
No	12	63.2

Self-reported familiarity with physician roles in the pharmaceutical industry was limited. The majority of respondents reported either minimal understanding or having heard of only one or two roles. Only a small proportion described themselves as very familiar with multiple roles (Table 2).

**Table 2:** Which of the following best describes your current familiarity with roles for physicians in the pharmaceutical industry?

Response	n	%
I am very familiar with multiple roles	3	15.8
I have heard of one or two roles	7	36.8
I have a minimal understanding	8	42.1
I am not familiar at all	1	5.3

When asked to identify a specific physician title within the pharmaceutical industry, the MSL role was most frequently selected. Awareness of other roles, such as Medical Director and Chief Medical Officer, was also selected, while a small percentage reported no awareness of any of the other listed roles (Table 3).

**Table 3:** Which of these titles are you currently aware of as potential roles for physicians in pharma? (Select one)

Role	n	%
Medical Science Liaison (MSL)	8	42.1
Medical Director	5	26.3
Chief Medical Officer	3	15.8
Drug Safety/Pharmacovigilance	1	5.3
None of the above	2	10.5
Medical Information	0	0
Vice President of Medical Affairs	0	0

Regarding the likelihood of considering a career in the pharmaceutical industry, nearly half of the respondents selected a neutral response. A smaller proportion indicated that they were "likely" or "very likely" to consider such a pathway, while a minority reported that they were "unlikely" or "very unlikely" (Table 4).

**Table 4:** How likely are you to consider pursuing an alternative clinical career in the pharmaceutical industry?

Response	n	%
Very likely	2	10.5
Likely	4	21.1
Neutral	9	47.4
Unlikely	3	15.8
Very unlikely	1	5.3

Perceived preparation by medical schools to explore non-clinical career pathways was low. More than half of respondents reported feeling "not at all" prepared to explore careers in the pharmaceutical industry, while only a small minority reported feeling "adequately" or "very well" prepared (Table 5).

**Table 5:** How well do you feel medical school has prepared you to explore non-clinical career options like those in the pharmaceutical industry?

Response	n	%
Very well	1	5.3
Adequately	3	15.8
Minimally	4	21.1
Not at all	11	57.9

Post-session findings

The post-session survey assessed changes in interest, understanding, and intent following participation in the mentor sessions.

Interest in learning more about physician roles within the pharmaceutical industry increased following the sessions. The majority of respondents reported being either "very interested" or "somewhat interested" in further exploration, while fewer reported "neutral" or "not very interested" (Table 6).

**Table 6:** After participating in these sessions, how interested are you in learning more about physician roles in the pharmaceutical industry?

Response	n	%
Very interested	4	36.4
Somewhat interested	4	36.4
Neutral	1	9.1
Not very interested	2	18.2
Not at all interested	0	0

All respondents reported increased understanding of physician roles within the pharmaceutical industry after the sessions. Most described themselves as "much more informed", while the remainder reported being "slightly more informed" (Table 7).

**Table 7:** How would you describe your current understanding of physician roles in the pharmaceutical industry compared to before the first session?

Response	n	%
Much more informed	7	63.6
Slightly more informed	4	36.4
About the same	0	0
Less informed	0	0

When asked which pharmaceutical physician role was most interesting following the sessions, the MSL role was selected by a majority of participants. Other roles, including Chief Medical Officer and Drug Safety/Pharmacovigilance, were also selected by a significant number of respondents (Table 8).

**Table 8:** Which of these roles now interests you most based on what you learned or discussed during the sessions? (Select one)

Role	n	%
Medical Science Liaison (MSL)	6	54.5
Chief Medical Officer	3	27.3
Drug Safety/Pharmacovigilance	1	9.1
None of the above	1	9.1
Medical Director	0	0
Medical Information	0	0
Vice President of Medical Affairs	0	0

A majority of respondents indicated that the sessions influenced their interest in non-clinical career pathways. A smaller proportion reported no change or uncertainty (Table 9).

**Table 9:** Did these sessions influence your interest in non-clinical career paths for physicians?

Response	n	%
Yes	6	54.5
No	2	18.2
Not sure	3	27.3

More than half of respondents reported being more likely to seek mentorship from professionals with experience in the pharmaceutical industry following participation in the sessions. The remainder reported no change or uncertainty (Table 10).

**Table 10:** Are you more likely to seek out mentors with experience in the pharmaceutical industry after this session?

Response	n	%
Yes	6	54.5
No	2	18.2
Not sure	3	27.3

## Discussion

This descriptive study evaluated whether structured mentor-focused sessions delivered within an immersive international educational program influenced medical students' and residents' awareness and interest in pharmaceutical industry careers, particularly the MSL role. The findings demonstrate limited baseline familiarity with pharmaceutical physician roles and suggest that even brief, structured exposure may increase participants' self-reported understanding and interest.

Baseline awareness and educational gaps

The pre-session findings revealed that a majority of participants had not previously considered a career in the pharmaceutical industry and that most reported limited familiarity with physician roles in Medical Affairs. More than half indicated that medical school had not prepared them to explore career pathways in the pharmaceutical industry. These findings align with broader literature demonstrating that traditional medical curricula primarily emphasize direct patient care trajectories, with comparatively limited formal exposure to non-traditional physician roles [1-5,11,15-21].

Prior research examining the MSL role internationally has demonstrated variability in awareness and understanding of the profession, even among HCPs [6,8,10]. Surveys conducted in other countries and regions have shown differences in familiarity with role responsibilities, metrics, and professional structures [6,8]. The baseline results observed in this study are consistent with these findings and suggest that similar awareness gaps may exist among medical trainees from diverse educational systems [1,2,5,16-18,20].

Post-session changes in awareness and interest

All post-session respondents reported increased understanding of the roles of pharmaceutical physicians, with the majority indicating they were "much more informed". Additionally, most participants reported being "somewhat" or "very interested" in learning more about pharmaceutical industry careers. More than half indicated that the mentor sessions influenced their interest in alternative clinical careers and increased their likelihood of seeking mentorship from someone working in the pharmaceutical industry.

Although the study design does not allow causal inference, these descriptive findings suggest that structured educational exposure may influence perceptions and stimulate further exploration. The MSL role emerged as the most frequently selected role of interest following the sessions. This may reflect alignment between the MSL role and competencies emphasized in medical training, including scientific literacy, evidence-based communication, and peer-to-peer dialogue [4,5,13].

Relevance of the MSL profession

The MSL profession has evolved significantly in recent decades, with increasing formalization of role expectations, performance metrics, and strategic integration within Medical Affairs departments [7,9-14]. The growing complexity of drug development, regulatory requirements, and scientific exchange has expanded the responsibilities of MSLs [6-9,11-14]. Literature describing key performance indicators, tracking systems, and benchmarking frameworks further demonstrates the maturation of the role within pharmaceutical organizations [7,9,11-14].

Given these developments, awareness of the MSL profession may be particularly relevant for trainees seeking scientifically focused roles outside traditional patient care settings. The emphasis on scientific exchange with HCPs and supporting informed clinical decision-making distinguishes the MSL role from commercial functions, such as sales representative positions, within pharmaceutical companies and should be emphasized when educating medical trainees about alternative physician career opportunities [4,10,16-20,22]. Physician careers within the pharmaceutical industry may involve professional, regulatory, and organizational challenges that differ from traditional clinical practice settings. Roles within Medical Affairs, such as MSL positions, often require working within evolving regulatory compliance frameworks, including Food and Drug Administration (FDA)-related requirements, as well as navigating complex corporate and cross-functional environments. Awareness of both the opportunities and potential challenges associated with these careers may help support more informed career decision-making among medical trainees.

Importance of structured mentorship

Mentorship serves a critical role in the formation of professional identity during medical training [15-20]. Exposure to diverse physician career pathways may broaden trainees' understanding of how medical expertise can be applied across healthcare systems. Structured mentor sessions provide a forum for discussing misconceptions, regulatory frameworks, ethical boundaries, and the patient-centered purpose of Medical Affairs roles.

The University of Utah School of Medicine Wilderness Medicine Program provides a distinctive context for such discussions. Immersive, high-intensity educational environments that emphasize leadership, adaptability, and interdisciplinary collaboration may foster openness to broader professional exploration. Embedding career-focused mentorship within this setting may enhance receptivity to discussions regarding alternative physician career pathways.

Implications for medical education

The findings highlight a potential gap in medical education regarding structured exposure to alternative clinical careers. As healthcare systems evolve and biotechnology innovation accelerates, physician roles within pharmaceutical and biotechnology organizations are likely to remain strategically important. Incorporating balanced, evidence-based discussions of such roles within medical curricula may support informed career decision-making.

The global diversity of participants in this study further suggests that awareness gaps may transcend individual educational systems. Structured exposure initiatives may therefore have international relevance.

Limitations

This study has several limitations. The sample size was limited, and participation in both mentor sessions and surveys was voluntary, which introduced potential selection bias. The post-session response rate was lower than the pre-session response rate. The surveys were anonymous and not linked to an individual, preventing paired analysis. The survey instrument was developed specifically for this educational evaluation and did not undergo formal psychometric validation. The mentor sessions were conducted by an individual with professional experience in Medical Affairs, including the MSL profession, which may have introduced presenter bias. Additionally, the study assessed immediate self-reported changes in awareness and interest rather than long-term outcomes, and no longitudinal follow-up was conducted to determine whether changes in career interest were sustained over time. The descriptive design precludes causal conclusions. Longitudinal follow-up across multiple cohorts would strengthen the understanding of sustained impact.

## Conclusions

This descriptive study evaluated whether structured mentor-focused sessions conducted during the University of Utah School of Medicine Wilderness Medicine Program influenced medical students' and residents' awareness of pharmaceutical industry careers, particularly the MSL profession. Baseline findings demonstrated limited familiarity with physician roles within Medical Affairs and limited perceived exposure to alternative clinical career pathways during medical training. Following participation in the mentor sessions, participants reported a greater understanding of physician roles in the pharmaceutical industry, increased interest in learning more about these careers, and a greater likelihood of seeking mentorship.

These findings suggest that even brief, structured educational exposure within immersive medical training environments may increase the awareness of alternative physician career pathways. The results also highlight a potential gap in traditional medical education regarding structured exposure to non-traditional physician roles and support the potential value of mentorship-based career exploration initiatives.

## Appendices

Description of the educational intervention

Title of Intervention

Mentor-Focused Educational Sessions on Alternative Clinical Careers with a Focus on Medical Science Liaisons

1. Overview of the Educational Intervention

This educational intervention consisted of structured, mentor-led sessions delivered during the University of Utah School of Medicine Wilderness Medicine Program held in Chamonix-Mont-Blanc, France, from June 30 through July 12, 2025. The purpose of the sessions was to introduce medical students and residents to alternative clinical career pathways within the pharmaceutical industry, with a particular focus on the Medical Science Liaison (MSL) role.

The sessions were optional and designed for participants interested in exploring non-traditional physician career paths beyond direct patient care.

2. Target Audience

The intervention was delivered to medical trainees participating in the program, including medical students and residents. Participants represented multiple institutions and six countries, reflecting a diverse and international cohort.

3. Learning Objectives

By the end of the sessions, participants were expected to describe alternative clinical career pathways available to physicians within the pharmaceutical industry, explain the role and responsibilities of MSLs, identify competencies and skills relevant to Medical Affairs careers, and evaluate their own interest in pursuing non-traditional clinical career pathways.

4. Educational Content

The educational content included an overview of physician career pathways within the pharmaceutical and biotechnology industries and an introduction to Medical Affairs as a function within the industry. Participants were also provided with a detailed overview of the MSL role, including scientific exchange with healthcare professionals, communication of clinical and real-world evidence, and the generation of insights to inform medical strategy. Additional discussion focused on differences between traditional clinical roles and industry-based positions, as well as common entry pathways and qualifications for MSL careers.

5. Instructional Methods and Format

The educational intervention consisted of two structured, in-person mentor-led educational sessions, each lasting approximately 45-60 minutes. The sessions included didactic presentations, interactive discussions, and question-and-answer components designed to encourage participant engagement and open dialogue regarding career exploration.

6. Delivery Setting

The sessions were conducted in person within the immersive environment of the Wilderness Medicine program in Chamonix-Mont-Blanc, France. The program setting provided a unique educational context that combined clinical training with broader career exploration opportunities.

7. Evaluation Methods

The effectiveness of the educational intervention was evaluated using anonymous pre- and post-session surveys administered electronically during the mentor sessions. Both surveys consisted of multiple-choice questions and Likert-type scale items. The pre-session survey was administered immediately prior to the mentor session, and the post-session survey was administered immediately after the session.

8. Outcome Measures

The surveys were designed to assess baseline awareness of pharmaceutical industry careers, prior exposure to non-traditional physician career pathways during medical training, changes in awareness of physician roles within the pharmaceutical industry, level of interest in learning more about these careers, and perceived attractiveness of specific roles, including the MSL profession.

9. Data Analysis

Survey responses were analyzed descriptively. Results were summarized using frequencies and percentages for each survey item. No inferential statistical analyses were performed due to the study's descriptive nature and the small sample size.

10. Reproducibility Statement

This educational intervention can be replicated in similar academic or training environments by implementing a structured, mentor-led session focused on alternative clinical careers, using comparable content, format, and evaluation methods as described above.
